# Fatigue behaviour of root filled teeth: The role of endodontic sealers and restorative techniques

**DOI:** 10.1111/eos.70077

**Published:** 2026-02-27

**Authors:** Andressa Weber Vargas, Rafaela Oliveira Pilecco, Pablo Machado Soares, Êmile de Moraes, Gabriel Kalil Rocha Pereira, Renata Dornelles Morgental

**Affiliations:** ^1^ Post‐Graduate Program in Oral Sciences Federal University of Santa Maria (UFSM) Santa Maria Rio Grande do Sul Brazil; ^2^ Department of Conservative Dentistry School of Dentistry Federal University of Rio Grande do Sul (UFRGS) Porto Alegre Rio Grande do Sul Brazil

**Keywords:** CAD/CAM, dental ceramics, fatigue, intraoral scanners, marginal/internal fit, milling machine

## Abstract

This in vitro study aimed to evaluate the fatigue behaviour of root canal treated teeth, filled with either AH Plus Jet (AHP) or AH Plus Bioceramic Sealer (AHPB) and rehabilitated with either mesio‐occlusal‐distal (MOD) restorations (direct resin composite) or endocrowns (indirect resin composite).

Seventy human upper premolars were prepared and randomly divided into five experimental groups according to the endodontic sealer and type of coronal restoration: AHP‐MOD: AHP sealer + MOD restoration; AHP‐EC: AHP sealer + endocrown; AHPB‐MOD: AHPB sealer + MOD restoration; AHPB‐EC: AHPB sealer + endocrown; control group (intact teeth). Monotonic testing was performed to define the fatigue parameters, then a cyclic fatigue test was conducted. Fracture analysis was performed using a stereomicroscope. Fatigue failure loads and the number of cycles to failure were analysed using the Kaplan–Meier and Mantel–Cox (Log Rank) test. Survival rates were calculated for each ‘step’ of the fatigue test. Fracture types were analysed descriptively. Teeth treated with AHP and restored with endocrowns achieved better results in both load resistance and number of cycles, compared to the AHP‐MOD group and teeth filled with AHPB regardless of the restoration used (*p* < 0.05). However, there was no significant difference when these groups were compared to the control (*p* > 0.05). Most failures (≥ 50%) were catastrophic (longitudinal fractures). Endodontically treated upper premolars filled with an epoxy resin‐based sealer and restored with endocrowns presented a superior fatigue behaviour, suggesting this combination may offer better long‐term outcomes compared to MOD restorations or calcium silicate‐based sealers.

## INTRODUCTION

Vertical root fracture (VRF) is a significant clinical concern, often leading to tooth extraction [1]. It is known that root canal preparation is one of the factors that favour VRF occurrence [2, 3, 4]. Therefore, endodontic obturation aims to seal the root canal with materials that reinforce the dental structure, in an attempt to increase the fracture resistance of teeth undergoing endodontic procedures [5, 6]. Currently, in endodontics, the most widely used obturation technique involves the combination of an endodontic sealer with a thermoplastic core material, such as gutta‐percha [7]. The sealer plays a crucial role in filling the interface between gutta‐percha and the dentinal walls, avoiding voids and marginal gaps, ultimately reducing the risk of microleakage [8].

In this context, endodontic sealers establish a connection between the filling material and the root canal walls, either through micromechanical anchorage or chemical adhesion [9, 10, 11]. Known as the “gold standard” for root canal filling, AH Plus Jet (Dentsply Sirona), an epoxy resin‐based sealer, has demonstrated good results in terms of adhesion to the root dentin [12]. In 2021, a tricalcium silicate sealer commercially available as AH Plus Bioceramic Sealer (Dentsply Sirona) was launched in the market with superior biological properties compared to its epoxy resin‐based counterpart [13]. In addition, bioceramic sealers offer other relevant properties, such as antibacterial capacity [14], adhesion to dentin through micromechanical penetration of the sealer [15], and the formation of a hydroxyapatite layer, which may form a mineral attachment to dentin surface [13, 16]. More recently, Quaresma et al. (2023) observed bond strength values for AH Plus Jet of 4.26 MPa, whereas AH Plus Bioceramic showed lower values of 1.19 MPa [15].

Beyond the role of obturation in the long‐term survival of endodontically treated teeth, which often exhibit moderate to extensive coronal structure loss, the restorative treatment remains a controversial and challenging issue [17]. The appropriate treatment choice depends on the extent and quality of the remaining dental structure [18]. A common option in daily clinical practice is the direct restoration of the remaining dental structure with resin composite, due to its feasibility and cost‐effectiveness. However, failures in extensive composite restorations, particularly those involving proximal areas, may occur, resulting from the fatigue of the fragile dental structure due to the propagation of microcracks under repeated loading [19].

To take advantage of the condition of endodontically treated teeth, another restorative option is the use of endocrowns. This technique results in macromechanical retention, generated by the walls of the pulp chamber in close contact with the restorative material, as well as micromechanical retention, induced by the use of adhesive bonding techniques for indirect restorations [20, 21]. This type of restoration has been recommended for posterior teeth, especially in cases of extensive loss of coronal structure associated with the presence of short, obliterated, curved, or weakened roots and in areas with limited interocclusal space, which would make reconstruction with prosthetic crowns and intraradicular retainers unfeasible [21]. Some previous studies have reported that premolars and molars rehabilitated with ceramic or composite resin endocrowns exhibit load to fracture values ranging from approximately 479.1 N to 1446.68 N for premolars, and from 2222.14 N to 3320.35 N for molars [18, 22, 23, 24, 25, 26].

Although the literature reports studies that have evaluated the fatigue resistance of endodontically treated teeth [25], to the best of our knowledge, no previous study has investigated the fatigue behaviour of root canal treated teeth filled with either AH Plus Jet (AHP) or AH Plus Bioceramic Sealer (AHPB) and rehabilitated with either mesio‐occlusal distal (MOD) direct resin composite restorations or indirect resin composite endocrowns. Therefore, the present study was conducted to evaluate the fatigue behaviour of endodontically treated teeth filled with AHP or AHPB sealers and restored with direct resin composite MOD restorations or indirect resin composite endocrowns. Additionally, the type of fracture was assessed among the different experimental and control groups. The null hypothesis was that there would be no statistically significant differences among these groups.

## MATERIAL AND METHODS

The manuscript of this laboratory study has been written according to ‘Preferred Reporting Items for Laboratory studies in Endodontology (PRILE) 2021′ guidelines [27] (Figure S1). The study was conducted under the approval of the local ethics and research committee (Protocol number 71,788,123.9.0000.5346).

### Sample selection

The sample consisted of seventy human upper premolars donated by the institutional Tooth Bank. The sample size calculation was based on the parameters of a previous study [28] and conducted in the G*Power software (v3.1, Heinrich‐Heine Universität), resulting in a total of 12 teeth per experimental group for the fatigue test (*n* = 60). Two more specimens from each group were included to perform the preliminary monotonic test (*n* = 10). The criteria orienting tooth selection included teeth with one or two roots, each necessarily containing two root canals, and intact or minimally restored crowns. In cases where the teeth had open apices, complex root anatomies, cracks, or fracture lines, they were excluded and replaced. To assess these criteria, teeth were radiographed using an intraoral digital sensor (RVG 5100; Carestream Health) in buccolingual (BL) and mesiodistal (MD) directions. Also, BL and MD widths in the coronal portion were measured with the aid of a digital calliper (Mitutoyo).

**FIGURE 1:**
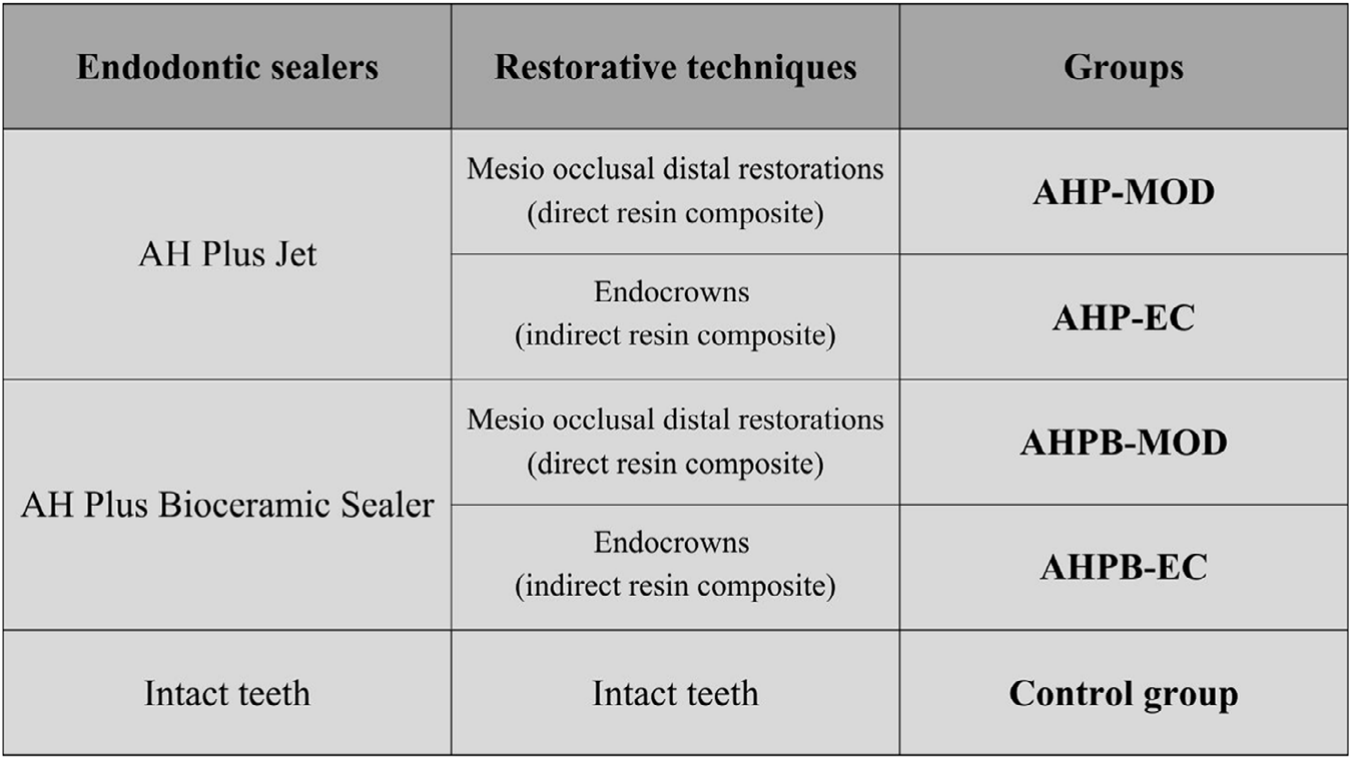
Schematic representation of the different experimental groups of the study, according to the type of endodontic sealer (AH Plus Jet or AH Plus Bioceramic Sealer) and the restorative strategy used (direct resin composite MOD restorations or indirect resin composite endocrowns).

Subsequently, the teeth were allocated into different experimental groups through stratified randomization, considering one or two roots and minor or major coronal widths, using the random.org software (www.random.org). The samples were divided into five groups, as follows: (1) AHP‐MOD: teeth filled with AHP and rehabilitated with MOD restorations (direct resin composite); (2) AHP‐EC: teeth filled with AHP and rehabilitated with endocrowns (indirect resin composite); (3) AHPB‐MOD: teeth filled with AHPB and rehabilitated with MOD restorations (direct resin composite); (4) AHPB‐EC: teeth filled with AHPB and rehabilitated with endocrowns (indirect resin composite); (5) Control group (intact teeth) (Figure 1).

### Root canal treatment

Endodontic access was performed using #1014 diamond burs (KG Sorensen) and Endo‐Z burs (Dentsply Sirona). Then, root canal patency was achieved with a #10 K‐file (Dentsply Sirona). The working length (WL) was determined 1 mm short of this measurement. All canals were prepared by the same operator (A.W.V.), using the R40 instrument (Reciproc; VDW) in an endodontic motor (X‐Smart Plus; Dentsply Sirona) in the “RECIPROC ALL” mode. Additionally, abundant irrigation of the root canals was performed using a 5 mL Luer Lock plastic syringe and a 27G needle (Ultradent Products Inc.), followed by aspiration with a disposable endodontic suction device (SSPlus). A total volume of 20 mL of 2.5% sodium hypochlorite (NaOCl) solution (Asfer Indústria Química) was used for each tooth.

After completing chemomechanical preparation, the final irrigation protocol was performed by agitating the irrigating solutions with the Easy Clean instrument (Easy Equipamentos) attached to a contra‐angle and micromotor (KaVo Dental) at 20,000 rpm. The instrument was kept 2 mm short of the WL and activated for 3 cycles of 20 s for each irrigant. The following solutions were used: 2 mL of 2.5% NaOCl, followed by 2 mL of 17% EDTA (Biodinâmica) and, again, 2 mL of 2.5% NaOCl. After the agitation protocol, the canals were irrigated with 5 mL of saline solution [29].

Finally, the canals were completely dried with absorbent paper points for teeth where the AHP sealer was used, and with just one absorbent paper point for teeth where AHPB was applied [30]. Obturation was performed using the respective sealer and the single‐cone technique. The gutta‐percha master point, corresponding to the last file used in canal preparation (R40), was inserted to the WL to verify its adaptation to the dentinal walls in the apical third and then selected or customized to fit. The AHP endodontic sealer was introduced into the root canal using a #25 K‐file [31], while the AHPB was placed using the applicator tip provided by the manufacturer, both to the WL. The gutta‐percha point, coated with endodontic sealer, was then inserted until it reached the WL. To assess radiographic quality of the obturation, specimens were radiographed in both BL and MD directions. Next, the coronal excess of gutta‐percha was removed with a heated Schilder 1–2 condenser (Odous de Deus). The same instrument was used, at room temperature, to condense the obturation material. The cervical portion of the canals was sealed with temporary restorative material (Cavit; 3 M ESPE), and all specimens were immediately stored in a moist environment at 37°C for 1 week to allow the sealers to set [32].

After completing the endodontic treatment, the teeth were embedded in plastic cylinders (25 mm in diameter and 20 mm in height) aligned with their long axis and positioned 2 mm below the cementoenamel junction using self‐curing acrylic resin (VIPI Flash; Vipi Produtos Odontológicos), a material that simulated the alveolar bone crest [33].

### MOD restoration

After endodontic treatment and embedding the teeth in the plastic cylinder, cavity preparation was carried out. The MOD inlay cavities were prepared using a diamond bur (#3131; KG Sorensen). The bur was mounted in a high‐speed handpiece, which was fixed to a modified microscope optical system to ensure parallelism along the tooth's long axis. The preparations had a bur convergence of approximately 10° and the following dimensions: a BL width of 3 mm, an occlusal box depth of 5 mm, and rounded internal line angles. Each diamond bur was used to prepare five teeth, and all preparations were finished using the same diamond bur (#3131) with a grit size of 25 µm [34].

Following cavity preparation, the dental structure (enamel and dentin) was conditioned with 37% phosphoric acid (Condac 37, FGM) for 15 s. The acid was thoroughly rinsed off using an air/water spray and the surface was dried with an air jet. Next, a universal adhesive system (Adhese; Ivoclar) was actively applied along the preparation for 20 s, dispersed with a gentle air jet, and light‐cured for 10 s. The MOD restorations were fabricated directly with resin composite (Tetric N‐Ceram, shade A3, Ivoclar) using the incremental technique with a light‐curing unit (power density 1200 mW/cm^2^—Radii Plus; SDI) for 20 s. The increments had a maximum thickness of 2 mm, and the same occlusal anatomy pattern was adopted. The final occlusal layer of the restoration, prior to light‐curing, was shaped using the piston employed for testing to replicate occlusal anatomy for fatigue testing. After this step, the restoration was light‐cured.

### Endocrown fabrication

The teeth were prepared to receive endocrown restorations by shaping the pulp chambers with a trunk‐shaped diamond bur (#3131; KG Sorensen) mounted in a high‐speed handpiece. The preparation depth corresponded to the active length of the diamond bur (approximately 4 mm), while the dimensions were adapted to each tooth's pulp chamber anatomy, using an angle of 6° and leaving an axial wall thickness of up to 2 mm [35].

Endocrown restorations were fabricated indirectly using a resin composite (Tetric N‐Ceram, shade A3, Ivoclar) with the incremental technique and a light‐curing unit (power density 1200 mW/cm^2^—Radii Plus; SDI) for 20 s. The increments had a maximum thickness of 2 mm, and the same occlusal anatomy pattern was adopted. As previously described, the final occlusal layer, which had not yet been light‐cured, was shaped with the piston used for testing to achieve the desired occlusal form. Then, the restoration was finally light‐cured.

Subsequently, the internal surface was air‐abraded with aluminium oxide (Polidental) at a distance of 10 cm with oscillating movements until the entire internal surface was treated. A primer (Monobond N, Ivoclar) was then applied for 15 s, left to react for 45 s, and subsequently dried with a brief air jet. The dental elements were conditioned (Primer A + Primer B, Ivoclar), and the resinous cement (Multilink N, Ivoclar) was mixed and applied to the restoration. Cementation was performed according to the manufacturer's instructions, using a constant and standardized load of 7.5 N. After these procedures, the excess of cement was removed with a disposable microbrush (KG Brush; KG Sorensen), and the assembly each surface was light‐cured for 20 s (power density 1200 mW/cm^2^—Radii Plus; SDI).

All specimens were stored in a humid environment at 37°C, for 24–72 h, until mechanical testing could be conducted.

### Fatigue testing

In order to obtain the fatigue test parameters, two specimens from each group were submitted to monotonic tests in a universal testing machine (EMIC DL1000) at 1 mm/min with the load applied by a 6 mm diameter stainless steel hemispherical piston. An adhesive tape (110 µm) was placed on the occlusal surface to enhance stress distribution and improve contact between the piston and the specimen [36]. During testing, the maximum load value recorded at the moment of fracture, corresponding to catastrophic failure of the material, was registered. Mean fracture load (in Newton) for each group was: AHP‐MOD: 1.253,94 N, AHP‐EC: 1.826,97 N, AHPB‐MOD: 1.312,5 N and AHPB‐ EC: 1.122,20 N.

Subsequently, the cyclic fatigue test was conducted at a frequency of 20 Hz, under distilled water at room temperature using an electrodynamic testing machine (Instron ElectroPuls E3000; Instron Corporation). The samples were placed on a metal platform at a 90° angle, where a cylindrical piston (6 mm in diameter) applied a load exclusively on the buccal and palatal cusp ridges. The initial fatigue load was defined as 10% of the mean fracture load obtained in the monotonic tests. The initial load was 100 N for 5000 cycles. The next step was 200 N for 10,000 cycles, followed by incremental steps increasing by 50 N each for 10,000 cycles until fracture occurred. Failure was identified by an abrupt drop in the applied load during cyclic loading, indicating loss of mechanical integrity. Once this sudden load decrease was detected, the test was immediately stopped. The fatigue failure load and the number of cycles for failure were recorded for each specimen [37].

### Mode of failure

Fracture analysis was performed after testing using a stereomicroscope (StereoDiscovery V20; Carl Zeiss Meditec) at ×10 magnification. Failures were classified as: Mode I, indicating small fractures in the tooth or inlay; Mode II, including one or more fractures of the cusp above the cement–enamel junction; or Mode III, showing longitudinal fracture damaging the tooth integrity. Modes I and II were considered reparable failures, while Mode III was classified as a catastrophic failure [34].

### Statistical analysis

All analyses were performed using the SPSS Statistics v.21 program (SPSS Inc.) at 5% significance level. The fatigue failure loads and the number of cycles to failure were analysed using the Kaplan–Meier and Mantel–Cox (Log Rank) test. Survival rates were calculated for each ‘step’ of the fatigue test. Fracture types were analysed descriptively.

## RESULTS

The AHP‐EC group displayed the greatest mean fatigue failure load (1267 N), while the AHPB‐MOD group showed the lowest one (970 N). The Kaplan–Meier and Mantel–Cox tests indicated that teeth treated with AHP and restored with endocrown restorations exhibited higher fatigue failure loads and longer survival, compared to the AHP‐MOD group and teeth filled with AHPB, regardless of the restoration used (*p* < 0.05). However, there was no significant difference when these groups were compared to the control (*p* > 0.05) (Table 1).

**TABLE 1:** Mean, standard deviation (SD), and confidence interval (CI 95%) for fatigue failure load (FFL), number of cycles for failure (CFF), and failure analysis for the different tested groups.

	FFL		CFF		Failure (%)		
Groups	Mean (SD)	CI 95%	Mean (SD)	CI 95%	Mode I	Mode II	Mode III
AHP‐MOD	1017^B^ (190)	912–1122	178.333^B^ (37.922)	115.000–235.000	2 (17%)	0	10 (83%)
AHP‐EC	1267^A^ (284)	1109–1424	228.333^A^ (56.779)	196.890–259.776	3 (25%)	3 (25%)	6 (50%)
AHPB‐MOD	970^B^ (168)	877–1063	169.000^B^ (33.552)	105.000–225.000	1 (9%)	2 (16%)	9 (75%)
AHPB‐EC	1010^B^ (300)	650–1600	177.000^B^ (60.024)	105.000–295.000	1 (8%)	1 (8%)	10 (84%)
CONTROL	1258^AB^ (425)	850–1750	197.333^AB^ (98.390)	94.079–300.588	0	4 (33%)	8 (67%)

*Note*: Different superscript letters in each column indicate statistical difference according to Kaplan–Meier and Log‐rank (Mantel–Cox) tests (*p* < 0.05).

**FIGURE 2:**
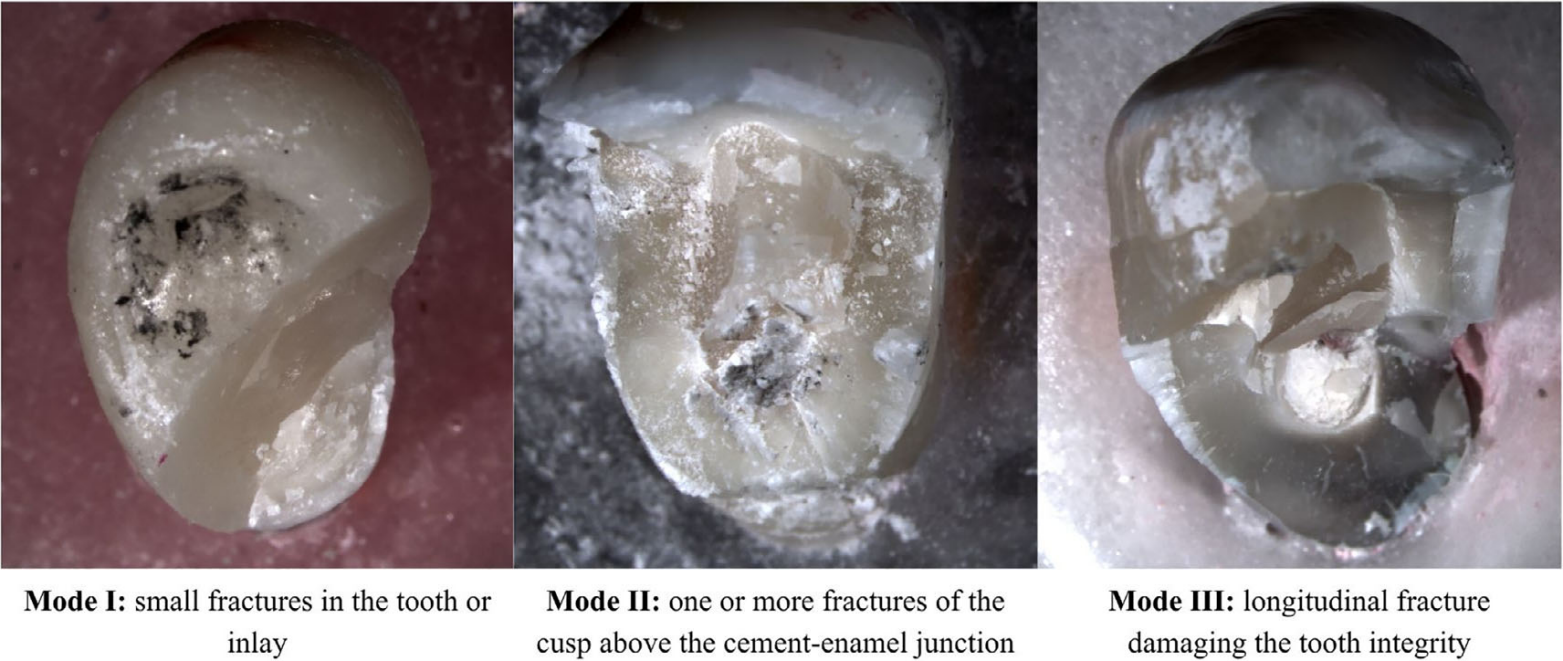
Representative images of the different failure modes observed in the experimental groups: Mode I (small fractures), Mode II (cusp fractures above the cemento‐enamel junction) and Mode III (longitudinal catastrophic fractures).

All groups showed most of their failures as longitudinal fractures, damaging the tooth integrity (Mode III), which was considered a catastrophic failure. Nonetheless, teeth filled with AHP and restored with endocrowns had a 50% chance of having repairable failures, whereas those restored with MOD had only a 17% chance (Table 1, Figure 2).

**FIGURE 3:**
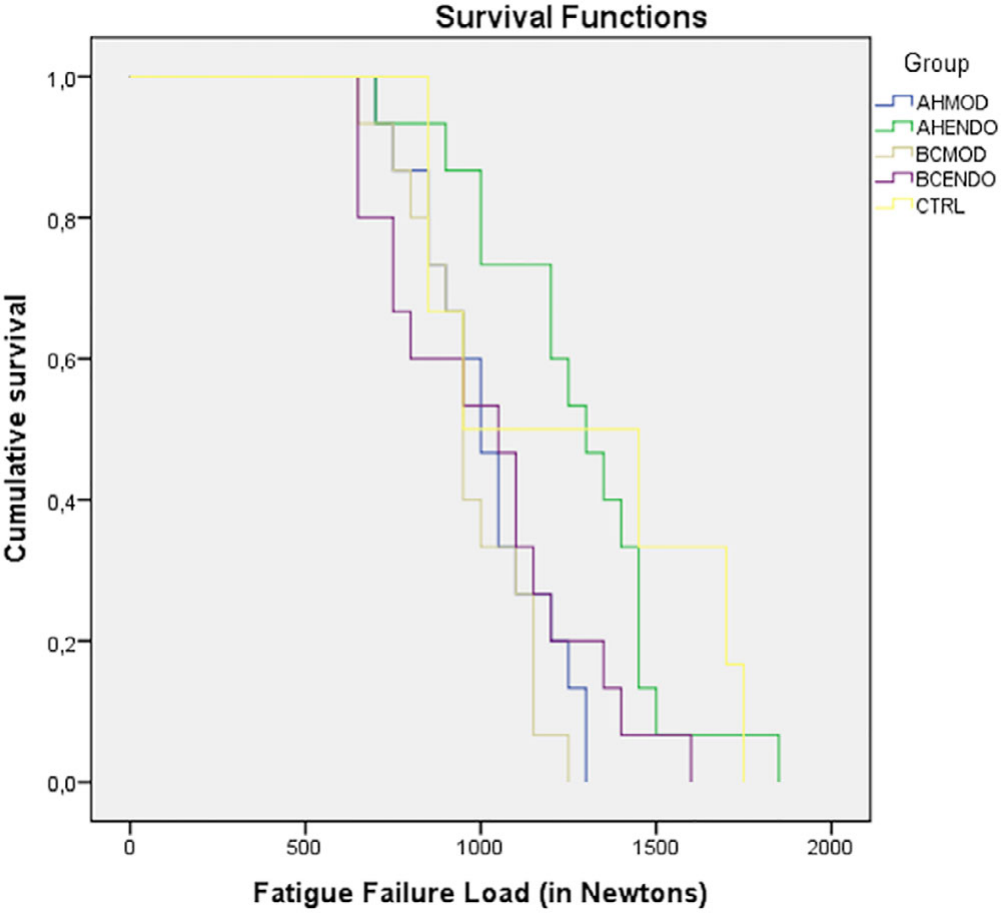
Survival plot of the different groups according to the fatigue test. Groups: AHMOD, AH Plus + MOD restoration; AHENDO, AH Plus + endocrown; BCMOD, AH Plus Bioceramic Sealer + MOD restoration; BCENDO, AH Plus Bioceramic Sealer + endocrown; CTRL, control.

Figure 3 shows the survival rate for the different groups in relation to the failure load, measured in Newtons. The survival rate for teeth treated with AHP and restored with endocrowns closely resembled that of the control group, in which no intervention was performed.

## DISCUSSION

This study evaluated the fatigue behaviour of endodontically treated teeth, filled with two endodontic sealers (AHP or AHPB) and rehabilitated with two restorative techniques (direct resin composite MOD restorations or indirect resin composite endocrowns). Given that the AHP‐EC group showed significantly better results than the other groups (AHP‐MOD, AHPB‐EC and AHPB‐MOD), the null hypothesis was rejected.

Root canal preparation may compromise tooth integrity, as mechanical instrumentation reduces root dentin thickness, while chemical irrigants contribute to dentin dehydration, leading to decreased modulus of elasticity and flexural strength [2, 4, 38]. In this context, root canal filling materials aim, among other objectives, to improve the mechanical behaviour of endodontically treated teeth [39]. Our results indicate that the use of AHP, an epoxy resin‐based sealer, resulted in increased fatigue behaviour compared to a calcium silicate‐based sealer, when endocrowns were used. This may occur due to the enhanced retention of the root canal filling material related to the mechanical interlocking between the epoxy resin and the canal walls, which may contribute to greater fracture resistance [40]. Past studies have confirmed this finding, although using different methodologies for fracture testing, such as static load tests [6, 28, 41]. In our study, we used the fatigue method, which, despite its limitations, is the most recommended approach to produce clinically relevant results [42].

Fractured teeth often result from cyclic fatigue, a phenomenon that occurs in the oral cavity in response to the stress generated during mastication. This test method enables the prediction of failures over time by effectively simulating the mechanical stimuli present in the oral environment [42, 43]. In these circumstances, fractures can occur with a load significantly lower than the tooth's maximum load capacity [42]. The cyclic fatigue test used in this study simulates, through controlled parameters such as the number of cycles, frequency, and load, the intermittent loading movements observed intraorally [43].

Restoring endodontically treated teeth with significant coronal loss remains a challenge in dental practice. One alternative restorative technique is the use of direct composite resin. However, in MOD restorations, there is a high prevalence of failures due to the fatigue of the fragile tooth structure from the propagation of microcracks under repeated loading [19], which corroborates our study's findings. Another option is the use of fibre posts [44], though their main advantage is limited to enhancing the retention of the core foundation. Also, intraradicular preparation for post placement may weakens the tooth structure and increases the risk of root fractures [45]. In the event of failure, in addition to exposing the tooth to irreversible fractures, the invasive nature of this type of restoration often excludes the possibility of subsequent interventions [46]. In this study, teeth filled with AHP and restored with endocrown had a 50% chance of having repairable failures, whereas those treated with MOD restorations had only a 17% chance; this may be due to the way this type of restoration distributes and supports the occlusal forces, as well as how the remaining tooth structure is used [25].

With the advancement of adhesive techniques, minimally invasive dentistry is challenging traditional post‐core concepts [47]. This approach is particularly applicable to endocrown restorations, which preserve more dental tissue, have reduced risk of catastrophic failures such as root fractures, root perforation and contamination of the root canal system, and involve fewer adhesive interfaces, resulting in lower costs [21]. Previous in vitro studies have identified endocrowns as a viable alternative for premolars [24, 48] supporting our findings. In addition, Belleflamme et al., in 2017, through a clinical retrospective study with molars, premolars and canines, found a 10‐year estimated survival rate of 98.8% in endocrowns performed in lithium‐disilicate glass‐ceramic and in polymer infiltrated ceramic network (PICN) material.

Standardization of human teeth for assessing fracture strength presents significant challenges due to anatomical variations, age and the timing of tooth extraction, all of which may influence the outcomes [49]. For extracted samples, it is crucial to standardize factors such as BL and MD widths at the coronal portion, as well as root canal morphology [27]. In our study, a millimetre gauge was employed to measure the BL and MD diameters at the coronal planes. Stratified randomization was carried out to ensure that root anatomies were evenly distributed across the sample.

It is important to note that the upper premolars used in this study were selected due to their high incidence of root fractures [50]. Furthermore, the single‐cone filling technique was chosen because it minimizes excessive dentin removal and eliminates the effects of finger spreaders used in the lateral condensation technique, as well as compactors in hot condensation [51]. Thus, the forces applied during root canal filling were neutralized, allowing only the effect of the filling materials to be evaluated. The single‐cone method was also selected for its simplicity, cost‐effectiveness and easy of reproduction [52].

One possible limitation of the present study is associated with the inherently lower mechanical performance of direct composite materials when compared with indirect composite systems, which may have influenced the biomechanical behaviour of the specimens. However, besides its lower cost, composite resin has interesting characteristics for endocrown fabrication due to its modulus of elasticity, which is similar to dentin, thus limiting irreparable fractures while maintaining high fracture resistance [53]. In addition, in the present study, a uniaxial vertical load was applied parallel to the long axis of the tooth to assess fracture resistance [54]. However, under actual oral conditions, loads and forces are applied from multiple directions. Another limitation of this study is the use of different restorative techniques among the experimental groups, involving direct and indirect procedures, which may have affected the biomechanical behaviour of the specimens. However, this study aimed to evaluate clinically feasible and practical restorative approaches. In addition, we acknowledge that the use of a 20 Hz frequency is considered relatively high, characterizing an accelerated fatigue test, which may have also influenced the results. However, this approach was intentionally adopted to allow the simulation of long‐term cyclic loading within a feasible experimental time. Moreover, the selected frequency was based on protocols previously described in the literature and widely used in in vitro fatigue studies [25, 37]. Furthermore, it is important to note that these are in vitro study results, and further studies, such as clinical trials, are needed to validate the reported findings.

## CONCLUSIONS

We can conclude that endodontically treated teeth filled with an epoxy resin‐based sealer and restored with endocrown‐type restorations presented a superior fatigue behaviour compared to MOD restorations or endodontically treated teeth filled with a calcium silicate‐based sealer, regardless of the type of coronal rehabilitation.

## AUTHOR CONTRIBUTIONS

**Conceptualization**: AW Vargas, GKRP Pereira and RD Morgental. **Data curation**: AW Vargas and RO Pilecco. **Investigation**: AW Vargas, PM Soares, GKR Pereira and RD Morgental. **Methodology**: AW Vargas, RO Pilecco, PM Soares, E Moraes, GKR Pereira and RD Morgental. **Software**: AW Vargas, RO Pilecco, GKR Pereira and RD Morgental. **Validation**: AW Vargas, RO Pilecco, GKR Pereira and RD Morgental. **Visualization**: AW Vargas, RO Pilecco, GKR Pereira and RD Morgental. **Project administration**: GKR Pereira and RD Morgental. **Resources**: GKR Pereira and RD Morgental. **Funding acquisition**: GKR Pereira and RD Morgental. **Supervision**: GKR Pereira and RD Morgental. **Formal analysis**: AW Vargas and RO Pilecco. **Writing—original draft**: AW Vargas. **Writing—review & editing**: AW Vargas, RO Pilecco, PM Soares, E Moraes, GKR Pereira and RD Morgental.

## CONFLICTS OF INTEREST STATEMENT

The authors declare no conflicts of interest.

## Supporting information



Supporting Information

## References

[1] Tamse A . Vertical root fractures in endodontically treated teeth: diagnostic signs and clinical management. Endodontic Topic. 2006;13(1):84–94. 10.1111/j.1601-1546.2006.00200.x

[2] Cohen S , Blanco L , Berman L . Vertical root fractures: clinical and radiographic diagnosis. J Am Dent Assoc. 2003;134(4):434–41. 10.14219/jada.archive.2003.0192 12733776

[3] Fuss Z , Lustig J , Tamse A . Prevalence of vertical root fractures in extracted endodontically treated teeth. Int Endod J. 1999;32(4):283–6. 10.1046/j.1365-2591.1999.00208.x 10551119

[4] Tavanafar S , Karimpour A , Karimpour H , Saleh AM , Saeed H . Effect of different instrumentation techniques on vertical root fracture resistance of endodontically treated teeth. J Dent. 2015;16(1 Suppl):50–5. PMC4476116 26106635

[5] Abdulsamad Alskaf MK , Achour H , Alzoubi H . The effect of bioceramic HiFlow and EndoSequence bioceramic sealers on increasing the fracture resistance of endodontically treated teeth: an in vitro study. Cureus. 2022;14(12), e33015. 10.7759/cureus.33051 36721549 PMC9883056

[6] Osiri S , Banomyong D , Sattabanasuk V , Yanpiset K . Root reinforcement after obturation with calcium silicate–based sealer and modified gutta‐percha cone. J Endod. 2018;44(12):1843–8. 10.1016/j.joen.2018.08.011 30384982

[7] Vishwanath V , Rao HM . Gutta‐percha in endodontics—a comprehensive review of material science. J Conserv Dent. 2019;22(3):216–22. 10.4103/JCD.JCD_420_18 31367101 PMC6632621

[8] Li GH , Niu LN , Zhang W , Olsen M , De‐Deus G , Eid AA et al. Ability of new obturation materials to improve the seal of the root canal system: a review. Acta Biomaterials. 2014;10(3):1050–63. 10.1016/j.actbio.2013.11.015 PMC3939610 24321349

[9] Donnermeyer D , Vahdat‐Pajouh N , Schäfer E , Dammaschke T . Influence of the final irrigation solution on the push‐out bond strength of calcium silicate‐based, epoxy resin‐based and silicone‐based endodontic sealers. Odontology. 2019;107(2):231–6. 10.1007/s10266-018-0392-z 30276580

[10] Huffman BP , Mai S , Pinna L , Weller RN , Primus CM , Gutmann JL . Dislocation resistance of ProRoot Endo Sealer, a calcium silicate‐based root canal sealer, from radicular dentine. Int Endod J. 2009;42(1):34–46. 10.1111/j.1365-2591.2008.01490.x 19125978

[11] Madhuri GV , Varri S , Bolla N , Mandava P , Akkala LS , Shaik J . Comparison of bond strength of different endodontic sealers to root dentin: An in vitro push‐out test. J Conserv Dent. 2016;19(5):461–4. 10.4103/0972-0707.190012 27656067 PMC5026108

[12] Saleh IM , Ruyter IE , Haapasalo M , Orstavik D . The effects of dentine pretreatment on the adhesion of root‐canal sealers. Int Endod J. 2002;35(10):859–66. 10.1046/j.1365-2591.2002.00585.x 12406381

[13] Sanz JL , López‐García S , Rodríguez‐Lozano FJ , Melo M , Lozano A , Llena C . Cytocompatibility and bioactive potential of AH Plus Bioceramic Sealer: an in vitro study. Int Endod J. 2022;55(10):1066–80. 10.1111/iej.13805 35950780 PMC9541143

[14] Hage W , Sarkis DK , Kallasy M , Sfeir G , Mallah M , El Hachem R . Antimicrobial activity of five calcium silicate‐based root canal sealers against a multispecies engineered biofilm: an in vitro study. J Contemp Dent Pract. 2023;24(9):707–14. 10.5005/jp-journals-10024-3556 38152946

[15] Quaresma SAL , Alves dos Santos GN , Silva‐Sousa AC , Camargo RV , Silva‐Sousa YT , Lopes‐Olhê FC . Influence of bioceramic cones on the quality of root canal filling relative to bond strength and adaptation of the adhesive interface. Clin Oral Investig. 2023;27(12):7919–33. 10.1007/s00784-023-05385-5 38032392

[16] Vallittu PK , Boccaccini AR , Hupa L , Watts DC . Bioactive dental materials—do they exist and what does bioactivity mean? Dent Mater. 2018;34(5):693–4. 10.1016/j.dental.2018.03.001 29571660

[17] Dotto L , Girotto LPS , Correa Silva Sousa YT , Pereira GKR , Bacchi A , Sarkis‐Onofre R . Factors influencing the clinical performance of the restoration of endodontically treated teeth: An assessment of systematic reviews of clinical studies. J Prosthet Dent. 2024;131(6):1043–50. 10.1016/j.prosdent.2022.03.030 35527069

[18] Biacchi GR , Mello B , Basting RT . The endocrown: an alternative approach for restoring extensively damaged molars. J Esthet Restor Dent. 2013;25(6):383–90. 10.1111/jerd.12065 24148141

[19] Drummond JL . Degradation, fatigue, and failure of resin dental composite materials. J Dent Res. 2008;87(8):710–9. 10.1177/154405910808700802 18650540 PMC2561305

[20] Bindl AD , Mörmann WH . Clinical evaluation of adhesively placed Cerec endo‐crowns after 2 years–preliminary results. J Adhes Dent. 1999;1(3):255–65. 11725673

[21] Belleflamme MM , Geerts SO , Louwette MM , Grenade CF , Vanheusden AJ , Mainjot AK . No post‐no core approach to restore severely damaged posterior teeth: An up to 10‐year retrospective study of documented endocrown cases. J Dent. 2017;63:1–7. 10.1016/j.jdent.2017.04.009 28456557

[22] Chang C‐Y , Kuo J‐S , Lin Y‐S , Chang Y‐H . Fracture resistance and failure modes of CEREC endo‐crowns and conventional post and core‐supported CEREC crowns. J Dent Sci. 2009;4(3):110–7. 10.1016/S1991-7902(09)60016-7

[23] Guo J , Wang Z , Li X , Sun C , Gao E , Li H . A comparison of the fracture resistances of endodontically treated mandibular premolars restored with endocrowns and glass fiber post‐core retained conventional crowns. J Adv Prosthodont. 2016;8(6):489–93. 10.4047/jap.2016.8.6.489 28018567 PMC5179488

[24] Lin C , Chang Y , Chang C , Pai C , Huang S . Finite element and Weibull analyses to estimate failure risks in the ceramic endocrown and classical crown for endodontically treated maxillary premolar. Eur J Oral Sci. 2010;118(1):87–93. 10.1111/j.1600-0722.2009.00704.x 20156270

[25] Ribeiro VF , Da Rosa LS , Tribst JPM , Bier CAS , Morgental RD , Valandro LF . Influence of height discrepancy between pulp chamber floor and crestal bone in the mechanical fatigue performance of endodontically‐treated teeth restored with resin composite endocrowns. J Mech Behav Biomed Mater. 2023;142:105854. 10.1016/j.jmbbm.2023.105854 37130494

[26] Altier M , Erol F , Yildirim G , Dalkilic EE . Fracture resistance and failure modes of lithium disilicate or composite endocrowns. Nigerian J Clin Pract. 2018;21(7):821–6. 10.4103/njcp.njcp_175_17 29984710

[27] Nagendrababu V , Murray PE , Ordinola‐Zapata R , Peters OA , Rôças IN , Siqueira JF Jr . PRILE 2021 guidelines for reporting laboratory studies in Endodontics: a consensus‐based development. Int Endod J. 2021;54(9):1482–90. 10.1111/iej.13542 33938010

[28] Sagsen B , Üstun Y , Pala K , Demirbuga S . Resistance to fracture of roots filled with different sealers. Dent Mater J. 2012;31(4):528–32. 22864204

[29] Paixão S , Rodrigues C , Grenho L , Fernandes MH . Efficacy of sonic and ultrasonic activation during endodontic treatment: a Meta‐analysis of in vitro studies. Acta Odontology Scandinavica. 2022;80(8):588–95. 10.1080/00016357.2022.2061591 35430959

[30] Nagas E , Cehreli ZC , Uyanik MO , Durmaz V . Dentin moisture conditions affect the adhesion of root canal sealers. J Endod. 2012;38(2):240–4. 10.1016/j.joen.2011.09.027 22244645

[31] Guinesi AS , Faria G , Tanomaru‐Filho M , Bonetti‐Filho I . Influence of sealer placement technique on the quality of root canal filling by lateral compaction or single cone. Braz Dent J. 2014;25(2):117–22. 10.1590/0103-6440201302370 25140715

[32] De Bem IA , de Oliveira RA , Weissheimer T , Bier CAS , Só MVR , Rosa RA . Effect of ultrasonic activation of endodontic sealers on intratubular penetration and bond strength to root dentin. J Endod. 2020;46(9):1302–8. 10.1016/j.joen.2020.06.014 32615175

[33] Bacchi A , Caldas RA , Schmidt D , Detoni M , Albino Souza M , Cecchin D . Fracture strength and stress distribution in premolars restored with cast post‐and‐cores or glass‐fiber posts considering the influence of ferule. Biomed Res Int. 2019;2019:2196519. 10.1155/2019/2196519 30719440 PMC6335778

[34] Pivetta Rippe M , Monaco C , Missau T , Wandscher VF , Volpe L , Scotti R . Survival rate and load to failure of premolars restored with inlays: An evaluation of different inlay fabrication methods. J Prosthet Dent. 2019;121(2):292–7. 10.1016/j.prosdent.2018.03.019 30093126

[35] Fages M , Bennasar B . The endocrown: a different type of all‐ceramic reconstruction for molars. J Can Dent Assoc. 2013;79:d140. 24309044

[36] Dartora G , Rocha Pereira GK , Varella de Carvalho R , Zucuni CP , Valandro LF Cesar PF . Comparison of endocrowns made of lithium disilicate glass‐ceramic or polymer‐infiltrated ceramic networks and direct composite resin restorations: fatigue performance and stress distribution. J Mech Behav Biomed Mater. 2019;100:103401. 10.1016/j.jmbbm.2019.103401 31445400

[37] Missau T , Venturini AB , Pereira GKR , Prochnow C , Valandro L , Rippe MP . Fatigue failure load of restored premolars: Effect of etching the intaglio surface of ceramic inlays with hydrofluoric acid at different concentrations. Oper Dent. 2018;43(2), E81–E91. 10.2341/16-345-L 29504886

[38] Uzunoglu E , Aktemur S , Uyanik MO , Durmaz V , Nagas E . Effect of Ethylenediaminetetraacetic Acid on root fracture with respect to concentration at different time exposures. J Endod. 2012;38(8):1110–3. 10.1016/j.joen.2012.04.026 22794216

[39] Karapinar Kazandag M , Sunay H , Tanalp J , Bayirli G . Fracture resistance of roots using different canal filling systems. Int Endod J. 2009;42(8):705–10. 10.1111/j.1365-2591.2009.01571.x 19467043

[40] Langalia AK , Dave B , Patel N , Thakkar V , Sheth S , Parekh V . Comparative evaluation of fracture resistance of endodontically treated teeth obturated with resin based adhesive sealers with conventional obturation technique: an in vitro study. J Int Oral Health. 2015;7(2):6–12. PMC4377154 25859099

[41] Topçuoǧlu HS , Tuncay Ö , Karataş E , Arslan H , Yeter K . In vitro fracture resistance of roots obturated with epoxy resin‐based, mineral trioxide aggregate‐based, and bioceramic root canal sealers. J Endod. 2013;39(12):1630–3. 10.1016/j.joen.2013.07.034 24238462

[42] Ordinola‐Zapata R , Fok ASL . Research that matters debunking the myth of the “fracture resistance” of root filled teeth. Int Endod J. 2021;54(3):297–300. 10.1111/iej.13479 33570814

[43] Missau T , Bello MC , Michelon C , Mastella Lang P , Kalil Pereira G , Baldissara P . Influence of endodontic treatment and retreatment on the fatigue failure load, numbers of cycles for failure, and survival rates of human canine teeth. J Endod. 2017;43(12):2081–7. 10.1016/j.joen.2017.07.013 29061355

[44] Zarow M , Devoto W , Saracinelli M . Reconstruction of endodontically treated posterior teeth–with or without post? Guidelines for the dental practitioner. Eur J Esthet Dent. 2009;4(4):312–27. 20111757

[45] Soares CJ , Santana FR , Silva NR , Preira JC , Pereira CA . Influence of the endodontic treatment on mechanical properties of root dentin. J Endod. 2007;33(5):603–6. 10.1016/j.joen.2007.01.016 17437882

[46] Rocca GT , Krejci I . Crown and post‐free adhesive restorations for endodontically treated posterior teeth: from direct composite to endocrowns. Eur J Esthet Dent. 2013;8(2):156–179. 23712338

[47] Magne P . Pascal Magne: “It should not be about aesthetics but tooth‐conserving dentistry”. Braz Dent J. 2012;213(4):189–91. 10.1038/sj.bdj.2012.769 22918368

[48] Lin C‐L , Chang Y‐H , Hsieh S‐K , Chang W‐J . Estimation of the failure risk of a maxillary premolar with different crack depths with endodontic treatment by computer‐aided design/computer‐aided manufacturing ceramic restorations. J Endod. 2013;39(3):375–9. 10.1016/j.joen.2012.11.042 23402510

[49] Marshall GW . Dentin: microstructure and characterization. Quintessence Int. 1993;24(9):606–17. 8272499

[50] Yamada Y , Tsubota Y , Fukushima S . Effect of restoration method on fracture resistance of endodontically treated maxillary premolars. Int J Prosthodont. 2004;17(1):94–8. 15008239

[51] Saw L‐H , Messer HH . Root strains associated with different obturation techniques. J Endod. 1995;21(6):314–20. 7673840 10.1016/S0099-2399(06)81008-3

[52] Heran J , Khalid S , Albaaj F , Tomson PL , Camilleri J . The single cone obturation technique with a modified warm filler. J Dent. 2019;89:103181. 10.1016/j.jdent.2019.103181 31430509

[53] Govare N , Contrepois M . Endocrowns: a systematic review. J Prosthet Dent. 2020;123(3):411–8. 10.1016/j.prosdent.2019.04.009 31353111

[54] Ghoneim AG , Lutfy RA , Sabet NE , Fayyad DM . Resistance to fracture of roots obturated with novel canal‐filling systems. J Endod. 2011;37(11):1590–2. 10.1016/j.joen.2011.08.008 22000470

